# Influence of maternal hyperglycemia on placental capillary distribution

**DOI:** 10.31744/einstein_journal/2024AO0583

**Published:** 2024-10-17

**Authors:** Jusciele Brogin Moreli, Natália Ferrari, Ana Maria Cirino Ruocco, Mariana Gomes de Oliveira Santos, Aline Rodrigues Lorenzon, Carla Patrícia Carlos, Marilza Vieira Cunha Rudge, Iracema de Mattos Paranhos Calderon

**Affiliations:** 1 Universidade Estadual Paulista "Júlio de Mesquita Filho Faculdade de Medicina de Botucatu Department of Gynecology and Obstetrics Botucatu SP Brazil Department of Gynecology and Obstetrics, Faculdade de Medicina de Botucatu, Universidade Estadual Paulista "Júlio de Mesquita Filho, Botucatu, SP, Brazil.; 2 Faculdade de Medicina de São José do Rio Preto Faculdade Ceres São José do Rio Preto SP Brazil Faculdade Ceres - Faculdade de Medicina de São José do Rio Preto, São José do Rio Preto, SP, Brazil.; 3 Huntington Medicina Reprodutiva-Eugin Group São Paulo SP Brazil Huntington Medicina Reprodutiva-Eugin Group, São Paulo, SP, Brazil.

**Keywords:** Placenta, Diabetes, gestational, *Diabetes mellitus*, type 1, *Diabetes mellitus*, type 2, Blood vessels, Hyperglycemia, Glycemic control, Glycemic Index

## Abstract

**Objective:**

This study was conducted to investigate the distribution of placental villous vessels in pregnant women with different degrees of hyperglycemia.

**Methods:**

A cross-sectional study was performed using placental samples from 30 pregnant women without diabetes (n=10), with gestational *diabetes mellitus* (n=10), and with previous diabetes (type 1 and type 2 diabetes; n=10). Maternal glycemic control was evaluated using the glycemic mean and glycated hemoglobin levels. Placental samples were obtained during elective cesarean sections and processed for villous vessel analysis using immunohistochemistry for Von Willebrand factor. Vessels within 10*μ*m of the villus margin were classified as peripheral, and vessels at a distance greater than 10*μ*m were classified as central. The number, area, and perimeter of all vessels were evaluated, and the relationship between vessel area and total area of placental villus was calculated.

**Results:**

Pregnant women with gestational *diabetes mellitus* and those with previous diabetes had higher glycated hemoglobin levels. The number of vessels was reduced in the villi of the previous Diabetes Group owing to peripheral reduction. Additionally, the area, perimeter, and percentage of peripheral blood were lower in the previous Diabetes Group than in the Non-Diabetic Group.

**Conclusion:**

Maternal glycemic levels can modify placental capillary distribution.

## In Brief

Moreli et al. demonstrated a reduction in vessels in the periphery of the placental villi in pregnant women with previous diabetes (type 1 and type 2). The placental vessels of this population are more distant from the maternal blood and may represent placental villous immaturity. These results were obtained when we classified the villous vessels as central or peripherial using 10μm of the villus margin as a reference.

## Highlights

■The number of vessels was reduced in the placental villi of pregnant women with diabetes.

■The placental villous vessels of women with previous diabetes were more distant from the maternal blood.

## Graphical abstract

**Figure:**
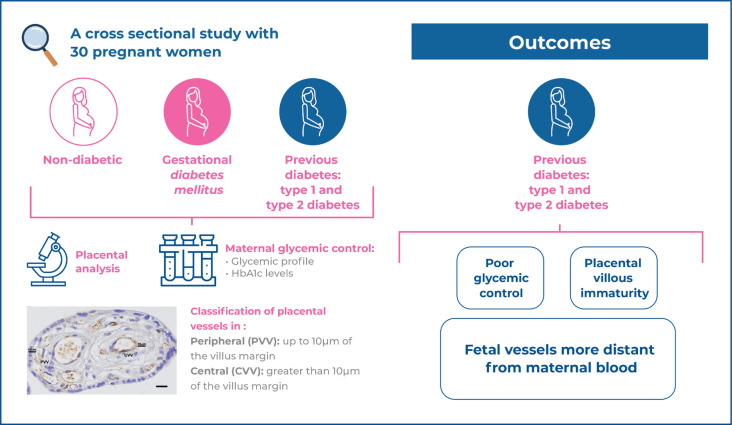

## INTRODUCTION

*Diabetes mellitus* (DM) [gestational DM (GDM), type 1 DM (DM1), and type 2 DM (DM2)] may occur concomitantly during pregnancy.^(1)^ Regardless of the classification, hyperglycemia is one of the most frequent clinical complications during pregnancy and is associated with high rates of macrosomia, hyperbilirubinemia, hypoglycemia, respiratory distress syndrome, shoulder dystocia, preterm birth, cesarean delivery, and perinatal mortality and morbidity.^(2)^ These complications may be because maternal glucose can cross the placenta, with consequent hyperglycemic exposure to the embryo/fetus during critical stages of intrauterine development.^(3)^ The pregnancy complications and poor fetal outcomes related to DM are associated with defects in placental development and function.^(4)^ This association is attributed to the placental position that separates the maternal and fetal circulation through the syncytiotrophoblast, which exposes the placenta to maternal circulation and the endothelium in contact with fetal blood.^(5)^

The human placenta is a highly vascularized organ by which respiratory gases, nutrients, and waste are exchanged between the maternal and fetal systems; it is responsible for embryo development and fetal growth.^(6–8)^ To ensure a successful pregnancy, the placenta depends on the adequate development of the fetal and uterine capillary networks, with sufficient plasticity to allow for the necessary changes throughout pregnancy.^(9)^ The formation and establishment of the placenta is one of the earliest factors for a successful pregnancy and until birth.^(6)^ In the last trimester of pregnancy, angiogenesis intensifies with the formation of new vessels through budding and of capillary loops through elongation.^(10)^

Maternal hyperglycemia alters placental development.^(11–12)^ Placentas exhibit characteristics, such as heavy weight,^(8–9)^ delayed villous maturation, chronic hypoxia, and chorangiosis.^(7,12)^ In addition, maternal glycemia appears to influence capillary formation, but not stromal or placental formation.^(13)^ In fact, blood vessels/villi were decreased in unexplained stillbirths compared to live births in women with diabetes,^(14)^ and the capillary volume and length were increased in the placentae of mothers with DM1 who had normally grown infants, compared to those without diabetes.^(13)^ According to Desoye et al.,^(5)^ the effect of hyperglycemia differs in the presence of GDM. In previous cases of diabetes, the placenta have had long-term effects, such as buffering excess maternal glucose or increasing vascular resistance. Diabetic insults at the later stages of gestation, such as those occurring in GDM, lead to short-term changes in various molecules involved in key functions.^(5)^

## OBJECTIVE

We aimed to investigate the distribution of placental villous vessels in pregnant women with different degrees of hyperglycemia by classifying the vessels as central (further away from the maternal blood) or peripheral (close to the maternal blood).

## METHODS

### Research design and participants

This study was conducted in the Diabetes and Pregnancy Service of *Faculdade de Medicina de Botucatu, Universidade Estadual Paulista "Júlio de Mesquita Filho* (protocol number 359/08) and in *Faculdade de Medicina Faceres* (CAAE: 50933221.8.1001.8083; #4.931.006) after approval by the Research Ethics Committee (REC) of both institutions. Written informed consent was obtained from all participants according to the principles of the Declaration of Helsinki.

This cross-sectional study evaluated the placental villous vessel parameters in 30 pregnant women. Pregnant women with previous diabetes [PD = DM1 (n=3) and DM2 (n=7)] were referred to the Diabetes and Pregnancy Service for a confirmed diagnosis. Gestational *diabetes mellitus* was diagnosed between the 24^th^ and 28^th^ gestational weeks using the 75g glucose tolerance test (GTT) according to the American Diabetes Association criteria 2023.^(1)^

In accordance with the American Diabetes Association (ADA) 2023,^(1)^ the GTT was performed in a one-step strategy with 75g glucose. After the intake of glucose, the plasma glucose was measured when the patient was fasting, at 1 and 2 hours at 24-28 weeks of gestation, in women not previously diagnosed with diabetes. Gestational *diabetes mellitus* diagnosis was made when the values were: fasting > 92mg/mL (5.1mmol/L), 1 hours >180mg/mL (10.0mmol/L), and 2 hours > 153mg/mL (8.5mmol/L).^(1)^

The inclusion criteria were as follows: (a) hyperglycemia defined at a maximum gestational age of 28 weeks for women with GDM; (b) prenatal and delivery care at the Diabetes and Pregnancy Service; (c) absence of clinically diagnosed infections and negative serology for HIV and syphilis, multiple pregnancies, fetal malformations, fetal death, or illicit drug habits; (d) signed and dated Research Consent Form; and (e) deliveries after the 36^th^ week of gestation. The patients were characterized by age, gestational age at delivery, mean glycemic level, HbA1c levels, smoking habits, and hypertension.

### Glycemic control and diabetes management

Patients in the PD Group received insulin therapy from the beginning of pregnancy. Glycemia in pregnant women with GDM was controlled through a combination of diet and exercise, and insulin therapy was administered according to medical conduct.

Patients with GDM and PD were subjected to glycemic profile^(14–16)^ with fasting, pre- and post-prandial glycemic levels for 24 hours at 2-week intervals until the 32^nd^ week, and weekly until delivery to follow the glycemic control and management of diabetes.^(17–19)^ The glycemic mean was calculated as the arithmetic mean of plasma glycemia levels in all glycemic profiles for the management of diabetes. In addition, the glycemic mean of the non-diabetic (ND) Group was calculated using glycemic levels measured during only one glycemic profile performed between the 24^th^ and 28^th^ gestational weeks. Plasma glucose levels were measured using the glucose oxidase method (Glucose Analyzer II, Beckman1, USA; CV = 10-800 mg/dL). Maternal blood samples were collected in Vacutainer tubes containing EDTA (Becton Dickinson1, USA) from the 36^th^ week of pregnancy to determine HbA1c levels using high-performance liquid chromatography (D10TM).

### Placental tissue samples

All placental samples were obtained from the cotyledons dissected from the medial placental zone and washed with ice-cold saline immediately after delivery. Areas with necrotic, hemorrhagic, or calcified lesions were excluded from the study. After this process, the maternal and fetal portions were dissected, and the fragments were fixed in 4% buffered paraformaldehyde, dehydrated in a growing series of ethanol, cleared in xylene, and embedded in paraffin blocks. The blocks were sectioned longitudinally in 4*μ*m sections, which were stretched in salinized slides for immunohistochemical reactions to mark factor VIII (AHF or anti-hemophilic globulin), a glycoprotein with a multimeric structure, synthesized exclusively by endothelial cells and megakaryocytes for better visualization of vessels.

### Immunohistochemical reaction

Immunohistochemical reactions were based on a polymer system linked to peroxidase. After deparaffinization and rehydration, the slides were submitted to antigen recovery in a 10mM citrate solution, pH 6.0, in a pressure chamber (Pascal, Dako^®^). Afterwards, endogenous peroxidase was blocked with an 8% hydrogen peroxide solution in methanol for 20 min, and binding with nonspecific proteins was blocked with 3% skim milk (Molico^®^) for 1 hour.

The slides were incubated with the primary antibody (Factor VIII, 1:2000, Abcam - code 61910^®^) and diluted in a specific solution (Dako^®^, Antibody Diluent with Background - code S3022) for 18 h in a humid chamber at 4^o^C. Subsequently, the secondary antibody linked to peroxidase (Advance Complex, Dako^®^-code K4069) was used, according to the manufacturer›s instructions. Moreover, the slides were stained with diaminobenzidine (DAB - Dako^®^, code K3468) and counter-stained with Mayer's hematoxylin (Merck^®^).

### Placental villous vessel analysis

Microscopic analysis of the slides was performed by a single-blind observer, who was not informed of the sample identification, using a computerized image analysis system of a Primo Star microscope (Carl Zeiss, Jena, Germany). The slides were examined at a lower magnification (200X) to identify the cut and then analyzed at a magnification of 400X.

**Figure 1:**
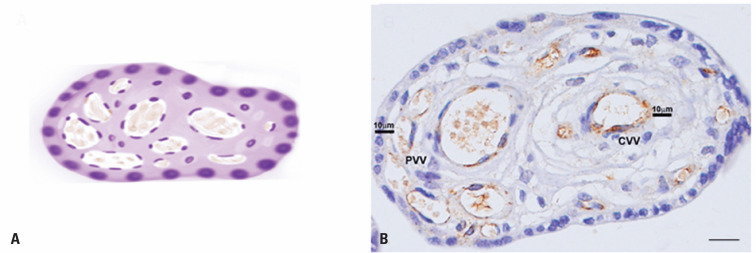
Placental villous vessel analysis example in a schematic figure (A) and photomicrography (B). The vessels were classified as peripheral (vessels within 10*μm of the vill*us margin) and central (vessels observed at a distance greater than 10*μ*m). The bars indicate 50*μ*m

Furthermore, on each slide, five fields were randomly selected for the analysis of villous vessels that were completely within their limits. First, the vessels were classified as peripheral (PVV; vessels within 10*μ*m of the villus margin) and central (CVV; vessels observed at a distance greater than 10*μ*m) (Figure 1A and 1B). After classification of the vessels, the quantity (number), area (*μ*m^2^), and perimeter (*μ*m) of all CVV and PVV were evaluated. Moreover, the relationship between the vessel area (PVV and CVV) and the total area of the placental villus was evaluated to define the percentage (%) of the vascular area in the villi.

### Statistical analysis

Analysis of variance and Tukey's multiple comparison test were used for normally distributed quantitative variables. For those with an abnormal distribution, a generalized linear model with gamma distribution, log-link function, and least squares (LS) means test were used for multiple comparisons. Statistical significance was set at 5% level (p<0.05).

## RESULTS

### Clinical data

**Figure 2:**
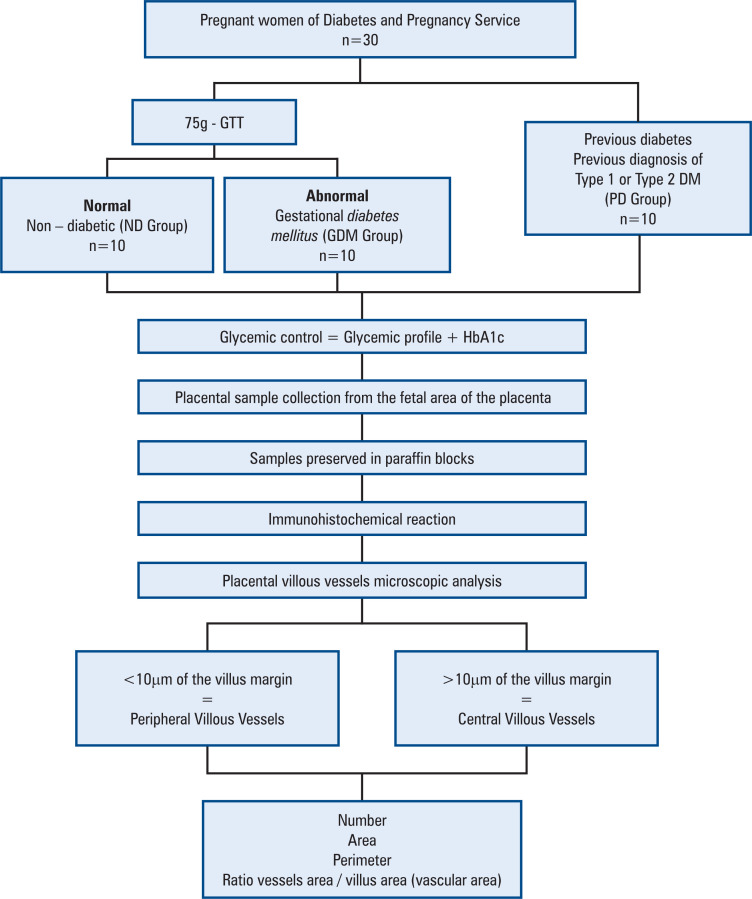
Research workflow

This study included 30 pregnant women at the Pregnancy Service of *Faculdade de Medicina de Botucatu* classified as ND, with GDM, and with PD. Each group comprised 10 pregnant women and their placentas that were analyzed according to the research workflow are presented in figure 2.

GTT: glucose tolerance test.

### Glycemic control

Except for glycemic control variables, maternal characteristics did not significantly differ among the groups (Table 1). Mean glycemic and HbA1c levels followed the same pattern in the GDM and PD Groups. These groups presented worse glycemic control than the ND Group (Table 1).

**Table 1:** Maternal characteristics

	ND Group (n=10)	GDM Group (n=10)	PD Group (n=10)	p value
Age (years), means±SD	29.70±5.45	30.40±6.05	30.45±3.54	0.62
GA (weeks), means±SD	38.50±1.60	37.84±1.36	37.20±0.26	0.06
Glycemic mean (mg/dL), means±SD	82.21±12.02#	110.58±7.0#	111.30±22.00#	<0.0001
HbA1c (%), means±SD	5.28±0.69	6.49±0.70#	6.98±1.28#	<0.0001
Smoking, n (%)	1.00 (10.00)	2.00 (20.00)	2.00 (20.00)	0.98
Hypertension, n (%)	1.00 (10.00)	4.00 (40.00)	4.00 (40.00)	0.07

Clinical data are presented as means±standard deviation or n (%).

# p<0.05 *versus* others groups.

GA: gestational age at delivery; HbA1c: glycated hemoglobin in the third trimester of pregnancy; SD: standard deviation; ND: non-diabetic; GDM: gestational *diabetes mellitus*; PD: previous diabetes.

### Placental villous vessel analysis

**Figure 3:**
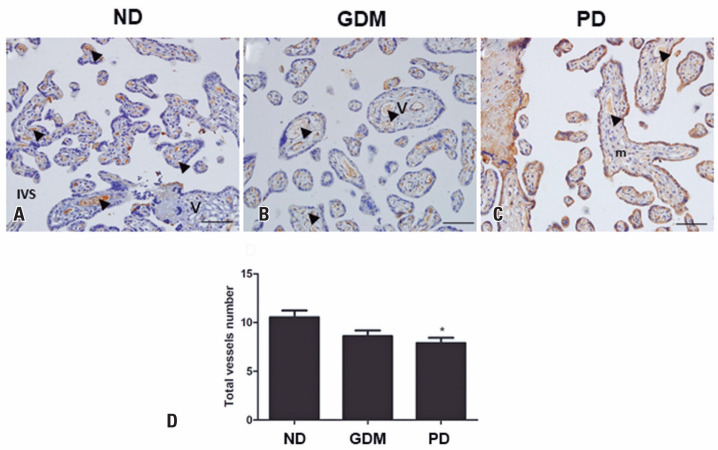
Placental total villous vessel analysis. Portions of intermediate villi in the placentas of non-diabetic (A), gestational *diabetes mellitus* (B), and previous diabetes (C) groups of pregnant women. Chorionic villi (v), mesenchyme (m), villous vessels (arrowhead), in the maternal-fetal exchange surface in contact with intervillous space. Note the immunolabeling for Von Willebrand factor - brown color in the endothelial cells of vessels. The bars indicate 50 *μ*m. 200X magnification

**Figure 4:**
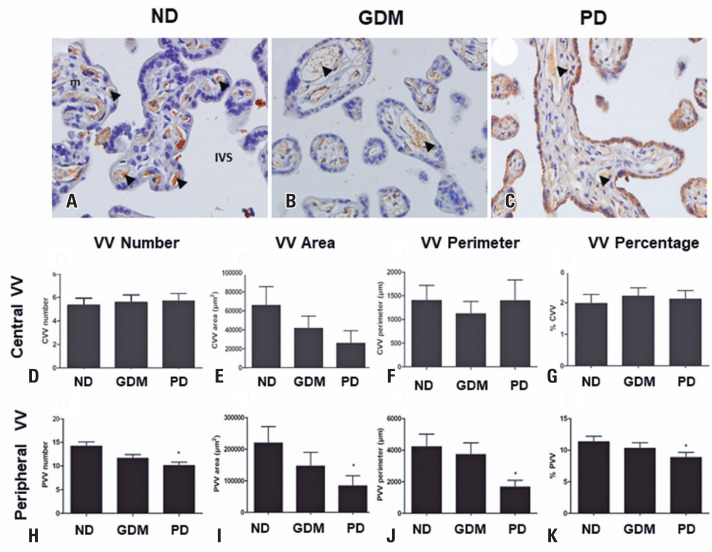
Placental central and peripheral villous vessels. Portions of intermediate villi in placentas of non-diabetic (A), gestational *diabetes mellitus* (B), and previous diabetes (C) groups of pregnant women. Note the immunolabeling for Von Willebrand factor - brown color in the endothelial cells of vessels. The bars indicate 50 *μ*m. 400X magnification. Central villous vessels analysis (D-G) and peripheral villous vessels analysis (H-K)

Placental intermediate villous vessel analysis of all vessels revealed a reduction in the PD Group compared to that in the ND Group (Figure 3). After this observation, the vessels were separated into the central and peripheral regions, and the analyses demonstrated a reduction only in the parameters of peripheral vessels (number, area, perimeter, and percentage of vessels) when compared with the ND Group. No differences were observed in the central vessel parameters (Figure 4).

* p<0.05 when compared to ND Group.

* p<0.05 when compared to ND Group.

## DISCUSSION

Despite being a common disease, the effects of DM have not been completely elucidated.^(20)^ The placental ability to adapt to a hyperglycemic environment affects pregnancy outcomes,^(5,21,22)^ particularly vascular modulation, which can change placental function with consequent alterations in fetal development.^(18,23,24)^ In this study, we further examined this condition by demonstrating that maternal hyperglycemia in pregnant women with diabetes can impact the migration of placental villous vessels to the periphery of the villus, an important placental adaptation at the end of pregnancy to ensure fetal growth.

According to a previous study by our group, the greatest number of villi and corresponding vessels in pregnant women with mild hyperglycemia provides a greater surface area for maternal-fetal exchange.^(18)^ In contrast, pregnant women with GDM or PD had the same villous surface area as pregnant women without diabetes but a smaller vascular surface area and a lower degree of capillarization.^(18)^

In this study, we used a new methodology to analyze the number and localization of villous vessels. The vessels more than 10*μm* from the villous margin were classified as central and were distant from the maternal blood in the intervillous space (placental membrane more ticker). The vessels found until 10*μm* of the villus margin were classified as peripheral and were close to the maternal blood that facilitates placental exchange. By applying this methodology, we observed that the number of vessels was reduced in the placental villi of the PD Group, owing to PVV reduction. In addition, the area, perimeter, and percentage of PVV were lower in the PD Group than in the ND Group. Therefore, placental fetal vessels are more distant from the maternal blood in women with hyperglycemia than in those without it, indicating placental villous immaturity.^(25)^

Wang et al.^(25)^ reported the following villus classifications: stem, terminal, mesenchymal, immature intermediate, and mature intermediate villi. Steam villi are condensed fibrous stroma that contain large vessels and microvessels and support the structures of the villous trees connected to the chorionic plate. Terminal villi have a high degree of capillarization and dilated sinusoids, and because of the vasculosyncytial membranes, it transfers electrolytes, O_2_, CO_2,_ and nutrients between the mother and the fetus.^(5,25)^

Mesenchymal villi are a primitive type of placental stroma composed of inconspicuous capillaries, cytotrophoblasts, and syncytiotrophoblast layers. They are important during the first week of pregnancy because of their endocrine functions. Immature intermediate villi are continuations of steam villi and contain reticular stroma, Hoffbauer cells, prominent vessels, and a discontinuous cytotrophoblast layer. This is considered the principal location of exchange during the first and second trimesters. Mature intermediate villi produce terminal villi, which are composed of fetal vascularization and a large exchange surface.^(5,25)^

Our results complement those of previous studies that demonstrated a smaller vascular surface area and lower degree of capillarization in placentas of women with PD.^(26)^ Angiogenesis in the placenta is regulated by the extracellular matrix and autocrine and paracrine signaling. Among these factors, vascular endothelial growth factor (VEGF) is highly expressed in terminal villous capillaries and adjacent trophoblasts and plays an important role in histopathological changes in placentas of women with diabetes.^(26,27)^ At the molecular level, PD placentas present similar patterns of VEGF and VEGF receptor (VEGFR) reactivity to those in normoglycemic placentas. In addition, women with GDM showed strong staining for VEGFR-1 in vascular and trophoblastic cells, whereas VEGF and VEGFR-2 were detected only in trophoblasts.^(23)^ Calderon et al. 2007^(18)^ hypothesized that the increase in glycemic levels, proportional to the severity of the maternal clinical condition and intrauterine hypoxia, inhibits villous angiogenesis and interferes with maternal-fetal exchanges, with a consequent risk of perinatal mortality.

The surface area, perimeter of blood vessels, and villus ratio have a significant effect on fetal development, as well as the mother's hyperglycemic status.^(28)^ Siassakos et al.^(29)^ analyzed 752 placental histopathology reports and suggested that the mechanism underlying stillbirth in placental distal villous immaturity is likely related to glucose dysmetabolism. Placentae from patients with uncontrolled diabetes tend to be heavy and exhibit immature villi, villous edema, fibrosis, excessive syncytial knot formation, and infarctions. In addition, fibrinoid necrosis, increased vasculosyncytial and syncytial basement membrane thickness, microvillous abnormalities, and vascular endothelial changes were observed.^(29)^

In contrast to previous studies, accelerated villous maturation and increased placental weight were significantly increased in placentas of pregnant women with diabetes after controlling for hypertension, and the neonates of the PD Group were more likely to be large for their gestational age and have a higher rate of preterm delivery.^(30)^ Using electron microscopy analysis, it was possible to demonstrate capillary structures of various sizes, both in free and stem villi, and they were observed to be denser in the GDM Group than in the control and type-1 DM Groups.^(31)^

## CONCLUSION

During a pregnancy complicated by diabetes, the human placenta undergoes several functional and structural pathological changes, including villous maturation defects. These findings reinforce the influence of glucose levels on the distribution of placental vessels, an indirect marker of organ maturation and development, and emphasize the importance of controlling maternal hyperglycemia during placental development and maturation to ensure fetal growth.
